# Fabrication and characterization of hexagonally patterned quasi-1D ZnO nanowire arrays

**DOI:** 10.1186/1556-276X-9-75

**Published:** 2014-02-12

**Authors:** Shou-Yi Kuo, Hsin-I Lin

**Affiliations:** 1 Department of Electronic Engineering, Chang Gung University, Kweishan, Taoyuan 333, Taiwan; 2 Advanced Optoelectronic Technology Center, National Cheng-Kung University, Tainan 701, Taiwan

**Keywords:** Zinc oxide, Sol–gel-derived ZnO thin films, Quasi-1D ZnO nanowire arrays

## Abstract

Quasi-one-dimensional (quasi-1D) ZnO nanowire arrays with hexagonal pattern have been successfully synthesized via the vapor transport process without any metal catalyst. By utilizing polystyrene microsphere self-assembled monolayer, sol–gel-derived ZnO thin films were used as the periodic nucleation sites for the growth of ZnO nanowires. High-quality quasi-1D ZnO nanowires were grown from nucleation sites, and the original hexagonal periodicity is well-preserved. According to the experimental results, the vapor transport solid condensation mechanism was proposed, in which the sol–gel-derived ZnO film acting as a seed layer for nucleation. This simple method provides a favorable way to form quasi-1D ZnO nanostructures applicable to diverse fields such as two-dimensional photonic crystal, nanolaser, sensor arrays, and other optoelectronic devices.

## Background

Zinc oxide (ZnO) has attracted much interest for its promising application in piezoelectric nanogenerators, gas sensors, light-emitting diodes, field-emission displays, and solar cells. Owing to its wide band-gap (3.37 eV at room temperature) and large exciton bonding energy of approximately 60 meV, ZnO has been recognized as an excellent candidate for short wavelength optoelectronic devices. Furthermore, ZnO nanostructures have many promising applications, such as lasers, light-emitting devices, and field emitters. Accordingly, a low-dimensional ZnO nanostructure might be used in novel nanodevices. Quasi-one-dimensional (quasi-1D) ZnO is one of the most important functional nanostructures, exhibiting transparent conductivity, piezoelectricity, and near-ultraviolet (UV) emission [1-3].

The growth of ZnO nanowires with precise control of their alignment, distribution, and aspect ratio is highly desirable for their potential applications in sensor arrays, high-efficiency photonic devices, near-UV lasers, and for assembling complex three-dimensional nanoscale systems [4-10]. A straightforward approach for this purpose is to fabricate metal nanoparticles, which are used as catalyst templates for the subsequent vapor–liquid-solid (VLS) growth of patterned nanowires [11]. In the past few years, numbers of approaches have been proposed to obtain nanoscale metal catalysts for the fabrication of patterned ZnO nanowire arrays, such as electron beam lithography (EBL), soft-photolithography, and mask lithography by porous alumina, self-assembled micro- or nanospheres [12-17]. EBL is known as a relatively complicated and costly method, thus unsuitable for large-scale fabrication. In contrast, imprint and nanosphere lithography (NSL) tend to be more promising as they are less costly techniques with a much higher throughput. Recently, several groups have reported the large-scale fabrication of ZnO nanowires using NSL technique [15-17]. However, the ZnO nanowires in these reports are either not nanopatterned or not truly vertically aligned. The limitation might result from the interconnection of the printed Au, un-optimized growth conditions and/or imperfect lattice matching between substrates and ZnO nanowires [15-17]. These drawbacks might hinder the consideration of such nanowire arrays from device applications. In addition, the VLS process is the most widely used technique for growing aligned ZnO, in which gold is the most frequently chosen metal catalyst [18-20]. However, as limited by the clean room requirements for silicon technology, gold is not the choice of metal for integrating with silicon. Therefore, it is important to explore a catalyst-free technique for ZnO nanowire growth.

In this paper, we report the catalyst-free synthesis of hexagonally patterned quasi-one-dimensional (quasi-1D) ZnO nanowire arrays with the assistance of NSL. The technique demonstrates an effective and economical bottom-up process for ZnO 1D nanostructures for applications as two-dimensional photonic crystals, sensor arrays, nanolaser arrays, and optoelectronic devices.

## Methods

**Figure 1:**
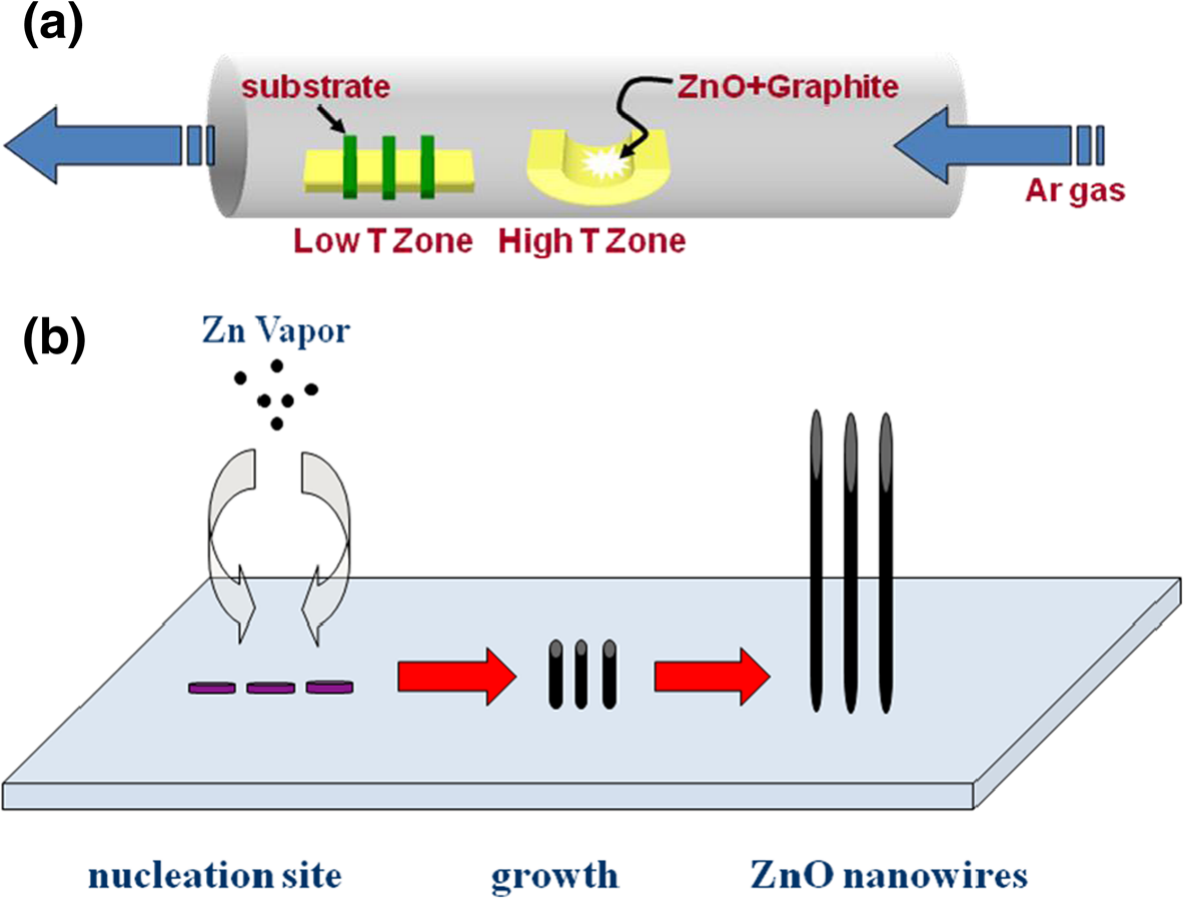
**Controlled growth of quasi-1D ZnO nanowires. (a)** Schematic diagram of experimental apparatus for growth of ZnO nanowires and **(b)** schematic illustration of growth mechanism for fabricating ZnO nanowire arrays.

The whole fabrication process and growth mechanism are schematically illustrated in Figure 1. First, aqueous solution of polystyrene (PS) nanospheres was diluted in methanol and spin-coated onto a silicon substrate. Afterward, the surface was covered with a ZnO film of approximately 200 nm thick via sol–gel process [21]. After the deposition, the film was inserted into a furnace and annealed in ambient atmosphere at 750°C for 1 h. By removing the PS spheres, a continuous hexagonal pattern was formed on the substrate. Growth of ZnO nanowires is performed inside a horizontal quartz tube. An alumina boat loaded with a mixture of ZnO + C (1:1) powder was placed at the center of the tube. Prior to heat treatment, the processing tube was evacuated to approximately 10^-3^ Torr by a rotary pump to eliminate the residual air in the tube. The furnace was heated to 850°C at a rate of 20°C/min and held at this temperature for 10 min, while we maintain the pressure of the system at 10 Torr with a constant Ar gas flow rate. The furnace was then switched off and cooled down to room temperature.

The morphologies and crystal structures of the resulting ZnO materials were characterized using field-emission scanning electron microscope (SEM) (Hitachi S-4300, Hitachi Co., Tokyo, Japan) and X-ray diffractometer (XRD) (BEDE Scientific Inc., Centennial, CO, USA). The optical property was studied by photoluminescence (PL) measurement (Jobin Yvon Triax320, Horiba Ltd., Minami-ku, Kyoto, Japan). The 325-nm line of a He-Cd laser was used as an excitation light source for the PL measurement.

## Results and discussions

**Figure 2:**
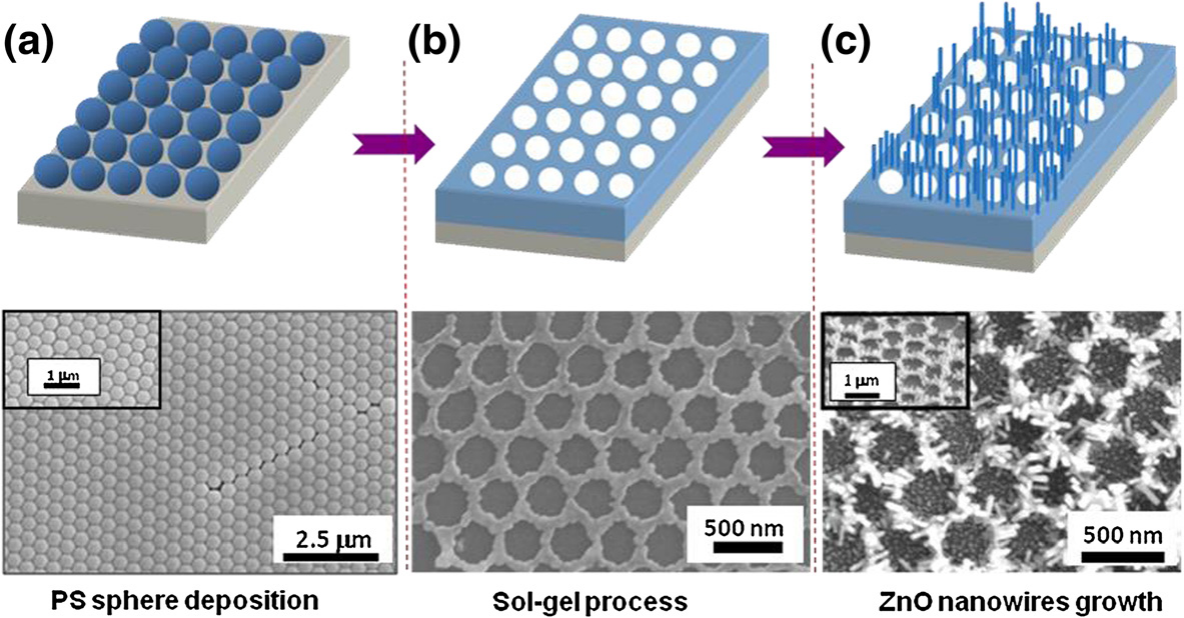
**SEM images.** Schematic illustration of the strategy for fabricating patterned quasi-1D ZnO nanowire arrays. Bottom of **(a)** shows low-magnification SEM image of the self-assembled monolayer polystyrene spheres. Inset is the high-magnification SEM image. Bottom of **(b)** reveals top-view SEM image of sol–gel-derived ZnO thin film patterned by periodic nanospheres. Bottom of **(c)** shows tilt-view SEM image of quasi-1D ZnO nanowire arrays grown on ZnO buffer layer, where the hexagonal pattern is apparent.

Figure 2a shows a typical SEM image of a PS nanosphere self-assembled monolayer on the substrate, indicating that a defectless region can be achieved. The ordering is reasonably good although point defects and stacking faults are observed in some areas, which may be produced by a variation in sphere size or process fluctuation. A closer examination presented in insert of Figure 2a shows perfectly ordered arrays. The self-assembled arrays of PS spheres were then used to guide ZnO growth onto substrate. For this purpose, sol–gel-derived ZnO thin films were spin-coated onto the self-assembled monolayer structure. According to previous studies, the annealing temperature of 750°C was chosen to be the post-thermal treatment parameter [21]. Due to the high liquidity of ZnO precursor, this technique produces a honeycomb-like hexagonal ZnO pattern, as shown in Figure 2b. It is clear that the honeycomb-like arrangement of the sol–gel-derived ZnO pattern was preserved during the growth process. Figure 2c presents a tilted SEM image of the obtained quasi-1D ZnO nanowire arrays.

**Figure 3:**
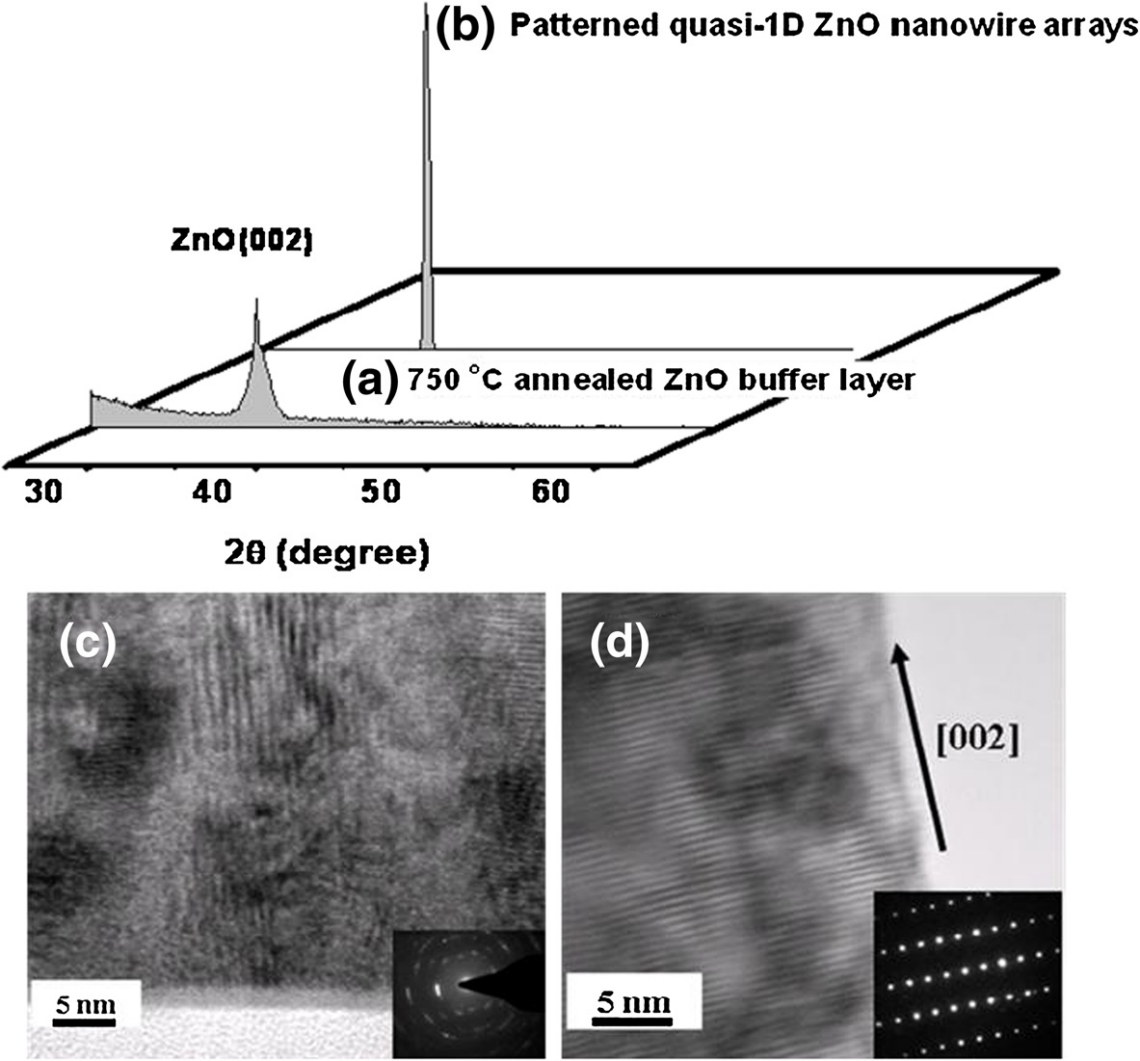
**XRD and SAED.** X-ray diffraction patterns of **(a)** sol–gel-derived ZnO thin film annealed at 750°C and **(b)** hexagonally patterned quasi-1D ZnO nanowire arrays. Both spectra show highly preferred *c*-axis growth. **(c)** and **(d)** are the electron diffraction patterns and HRTEM images of sol–gel-derived ZnO layer and ZnO nanowire, respectively.

Figure 3 curve a shows the XRD pattern of sol–gel-derived ZnO thin films annealed at the temperatures of 750°C. The typical thickness of ZnO films is 200 nm, which was determined from the cross-sectional SEM images. The XRD spectra reveal that the ZnO films developed without the existence of secondary phases and clusters, and only the ZnO (002) diffraction plane is observed. The *c*-axis orientation in ZnO films might be due to a self-texturing mechanism as discussed by Jiang et al*.*[22]. The aligned ZnO nanowires were then grown using the pattern in a tube furnace at elevated temperature. A honeycomb-like pattern of dense and well-aligned ZnO nanowire arrays was produced as shown by the SEM image in Figure 2c. For a growth time of 10 min, the length of the ZnO nanowires was approximately 100 nm and their diameters ranged from 20 to 30 nm. Figure 3 curve b shows the XRD pattern of the patterned quasi-1D nanowire arrays. It was found that the results prior to and after the growth of nanowires show no significant difference. The fact that no additional peaks appearing in the XRD spectra strongly supports the good alignment of the ZnO nanowires along the hexagonal *c*-direction. As expected, the highly enhanced (002) peaks can be seen as a result of the vertical orientation of the ZnO nanowires. Shown in Figure 3c,d are the electron diffraction pattern and high-resolution transmission electron microscope (HRTEM) images of annealed ZnO film and patterned ZnO nanowire, respectively. These results indicate a good crystallinity of the 1D ZnO nanowire, which is consistent with the XRD results. The HRTEM image also indicates the nanowires preferentially grow along the [002] direction (*c*-axis). This emphasizes the belief that the ZnO buffer layers are much more advantageous substrates for the fabrication of highly ordered ZnO nanostructures.

**Figure 4:**
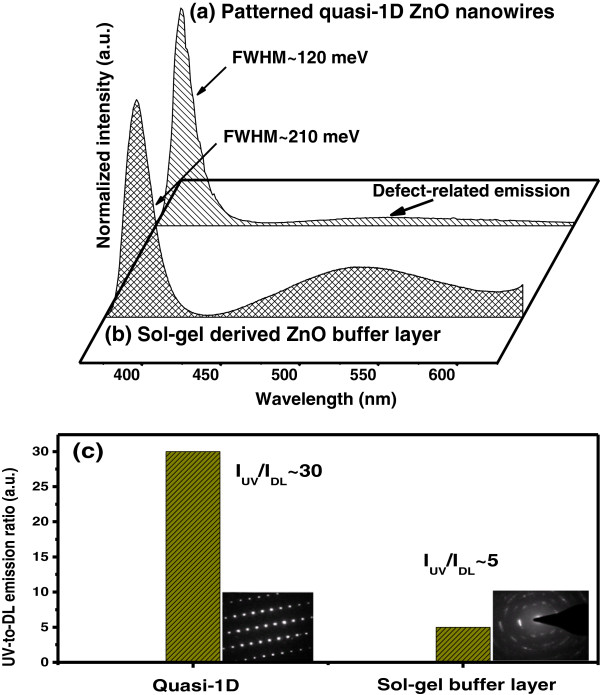
**PL spectra.** Room-temperature PL spectra of **(a)** the hexagonally patterned ZnO nanowire arrays and **(b)** ZnO buffer, respectively. Two peaks attributed to extionic recombination (*I*_UV_) and defect-related emission (*I*_DL_) are clearly seen. **(c)** The variation of UV-to-DL emission intensity ratio (*I*_UV_/*I*_DL_) of ZnO samples.

The PL spectra of the patterned ZnO nanowire arrays and buffers are illustrated in Figure 4 curves a and b, respectively. The emission consists of two main parts: a strong UV emission located at approximately 3.2 eV and a much weaker deep level (DL) related emission located at approximately 2.4 eV. According to the SEM measurements, the thickness of the buffer layer and the diameter of the nanowire are approximately 200 and approximately 50 nm, respectively. On average, the diameter is much larger than the exciton Bohr radius (approximately 2*.*34 nm) in bulk ZnO. Therefore, there is no significant blue shift according to the quantum confinement effect in the PL spectrum. Figure 4c reveals the variation of UV-to-DL emission intensity ratio (*I*_UV_/*I*_DL_) of patterned quasi-1D ZnO nanowires and sol–gel-derived ZnO buffer layer. The high UV-to-DL emission intensity ratio (*I*_UV_/*I*_DL_ approximately 30) and small FWHM (approximately 120 meV) of the UV peak confirm its high crystal and optical quality. The UV emission is attributed to the near-band-edge (NBE) exciton emission, and the DL emission is most commonly regarded as coming from the singly ionized oxygen vacancies or surface states. The intensity ratio of the NBE to the DL emission in quasi-1D nanowire arrays is larger than that of sol–gel-derived ZnO buffer layer, which indicates there are more oxygen vacancies for the sample grown at low temperature. This result agrees well with the prediction above.

Based on the above experimental results, we found that the ZnO thin films with *c*-axis preferred orientation will provide nuclei sites for the further growth of the nanowires through self-catalyst process [23]. According to the low energy principle, the [0001] plane is the fastest growing crystallographic plane [24]. Therefore, ZnO nanowires are high *c*-axis orientation. In addition, density control of ZnO nanowire arrays is a valuable concern in the research of field-emitter and photovoltaic devices. In this study, the annealed sol–gel-derived ZnO thin films were used as substrates to fabricate ZnO nanowire arrays. Compared to those unannealed ZnO thin films, the density of nanowire arrays becomes larger and more homogeneous. Recently, Liao et al. also proposed that the residual stresses in the thin film and the density of the nanowire array are in inverse proportion, and will have potential applications in modifying the density of ZnO nanowire arrays [25]. The intensity ratio of the NBE to the DL emission in honeycomb-like nanowires is larger than sol–gel-derived films, which indicates there are more oxygen vacancies for the sample grown at low temperature. This result indicates the proposed simple method is cost-effective approach to fabricated quasi-1D ZnO nanostructures with high-quality optical property.

## Conclusions

In summary, we have fabricated hexagonally patterned quasi-1D ZnO nanowire arrays through simple chemical methods. Instead of using metal catalyst, sol–gel-derived ZnO thin film was used as the periodic nucleation sites for nanowire growth with the aid of a PS nanosphere SAM. Structural and optical measurements demonstrate that the quasi-1D nanowires possess high quality. By observation of the process of ZnO nanowire growth, a vapor transport solid condensation mechanism was proposed, in which the role of ZnO thin film was to provide nucleation sites for nanowire growth. The technique is a self-catalyzed process that is entirely bottom-up and can be effectively scaled up to the fabrication of ZnO photonic crystal devices.

## Abbreviations

NBE: Near-band-edge; PL: Photoluminescence; SAED: Selected area electron diffraction; TEM: Transmission electron microscope; XRD: X-ray diffraction.

## Competing interests

The authors declare that they have no competing interests.

## Authors’ contributions

HIL designed and carried out the experiment, statistical analysis, and participated in the draft of the manuscript. SYK supervised the research and revised the manuscript. Both authors read and approved the final manuscript.

## References

[B1] Kim DC Kong BH Cho HK Morphology control of 1D ZnO nanostructures grown by metal-organic chemical vapor deposition J Mater Sci Mater Electron 2008 9 760 763 10.1007/s10854-007-9404-4

[B2] Service RF Will UV lasers beat the blues? Science 1997 9 895 10.1126/science.276.5314.895

[B3] Kong XY Wang ZL Spontaneous polarization-induced nanohelixes, nanosprings, and nanorings of piezoelectric nanobelts Nano Lett 2003 9 1625 1631 10.1021/nl034463p

[B4] Arnold MS Avouris P Pan ZW Wang ZL Field-effect transistors based on single semiconducting oxide nanobelts J Phys Chem B 2003 9 659 663 10.1021/jp0271054

[B5] Huang MH Mao S Feick H Yan H Wu Y Kind H Weber E Russo R Yang P Room-temperature ultraviolet nanowire nanolasers Science 2001 9 1897 1899 10.1126/science.1060367 11397941

[B6] Liu C Zapien JA Yao Y Meng X Lee CS Fan S Lifshitz Y Lee ST High-density, ordered ultraviolet light-emitting ZnO nanowire arrays Adv Mater 2003 9 838 841 10.1002/adma.200304430

[B7] Bai XD Wang EG Gao PX Wang ZL Measuring the work function at a nanobelt tip and at a nanoparticle surface Nano Lett 2003 9 1147 1150 10.1021/nl034342p

[B8] Yi GC Wang C Park WII ZnO nanorods: synthesis, characterization and applications Semicond Sci Technol 2005 9 22 10.1088/0268-1242/20/4/003

[B9] Li L Zhai T Zeng H Fang X Bando Y Golberg D Polystyrene sphere-assisted one-dimensional nanostructure arrays: synthesis and applications J Mater Chem 2011 9 40 56 10.1039/c0jm02230f

[B10] Ramírez D Gómez H Lincot D Polystyrene sphere monolayer assisted electrochemical deposition of ZnO nanorods with controlable surface density Electrochim Acta 2010 9 2191 2195 10.1016/j.electacta.2009.11.055

[B11] Wagner RS Ellis WC The vapor–liquid–solid mechanism of crystal growth and its application to silicon Trans Metall Soc AIME 1965 9 1053 1064

[B12] Ng HT Han J Yamada T Nguyen P Chen YP Meyyappan M Single crystal nanowire vertical surround-gate field-effect transistor Nano Lett 2004 9 1247 1252 10.1021/nl049461z

[B13] Greyson EC Babayan Y Odom TW Directed growth of ordered arrays of small-diameter ZnO nanowires Adv Mater 2004 9 1348 1352 10.1002/adma.200400765

[B14] Chik H Liang J Cloutier SG Kouklin N Xu JM Periodic array of uniform ZnO nanorods by second-order self-assembly Appl Phys Lett 2004 9 3376 3378 10.1063/1.1728298

[B15] Wang X Summers CJ Wang ZL Large-scale hexagonal-patterned growth of aligned ZnO nanorods for nano-optoelectronics and nanosensor arrays Nano Lett 2004 9 423 426 10.1021/nl035102c 25427146

[B16] Rybczynski J Banerjee D Kosiorek A Giersig M Ren ZF Formation of super arrays of periodic nanoparticles and aligned ZnO nanorods - simulation and experiments Nano Lett 2004 9 2037 2040 10.1021/nl048763y

[B17] Banerjee D Rybczynski J Huang JY Wang DZ Dempa D Ren ZF Large hexagonal arrays of aligned ZnO nanorods Appl Phys A 2005 9 749 752

[B18] Song J Wang X Riedo E Wang ZL Systematic study on experimental conditions for large-scale growth of aligned ZnO nanowires on nitrides J Phys Chem B 2005 9 9869 9872 10.1021/jp051615r 16852193

[B19] Wang XD Song JH Li P Ryou JH Dupuis RD Summers CJ Wang ZL Growth of uniformly aligned ZnO nanowire heterojunction arrays on GaN, AlN, and Al0.5Ga0.5N Substrates J Am Chem Soc 2005 9 7920 7923 10.1021/ja050807x 15913382

[B20] Wang XD Song JH Summers CJ Ryou JH Li P Dupuis RD Wang ZL Density-controlled growth of aligned ZnO nanowires sharing a common contact: a simple, low-cost, and mask-free technique for large-scale applications J Phys Chem B 2006 9 7720 7724 10.1021/jp060346h 16610866

[B21] Kuo SY Chen WC Lai FI Cheng CP Kuo HC Wang SC Hsieh WF Effect of doping concentration and annealing temperature on properties of highly-oriented Al-doped ZnO films J Crystal Growth 2006 9 78 84 10.1016/j.jcrysgro.2005.10.047

[B22] Jiang X Jia CL Szyszka B Manufacture of specific structure of aluminum-doped zinc oxide films by patterning the substrate surface Appl Phys Lett 2002 9 3090 3092 10.1063/1.1473683

[B23] Ham H Shen G Cho JH Lee TJ Seo SH Lee CJ Vertically aligned ZnO nanowires produced by a catalyst-free thermal evaporation method and their field emission properties Chem Phys Lett 2005 9 69 73 10.1016/j.cplett.2005.01.084

[B24] Hu JQ Bando Y Growth and optical properties of single-crystal tubular ZnO whiskers Appl Phys Lett 2003 9 1401 1403 10.1063/1.1558899

[B25] Liao X Zhang X Li S The effect of residual stresses in the ZnO buffer layer on the density of a ZnO nanowire array Nanotechnology 2008 9 225303 10.1088/0957-4484/19/22/225303 21825758

